# Baseline self-efficacy predicts subsequent engagement behavior in an online physical activity intervention

**DOI:** 10.3389/fspor.2024.1401206

**Published:** 2024-07-03

**Authors:** Seungmin Lee, Nicholas D. Myers, André G. Bateman, Isaac Prilleltensky, Adam McMahon, Ahnalee M. Brincks

**Affiliations:** ^1^ Division of Health and Wellness Studies, Binghamton University, Binghamton, NY, United States; ^2^ Department of Kinesiology, Michigan State University, East Lansing, MI, United States; ^3^ Department of Sociology, Psychology and Social Work, The University of the West Indies at Mona, Mona, Kingston, Jamaica; ^4^ School of Education and Human Development, University of Miami, Coral Gables, FL, United States; ^5^ Human Development and Family Studies, Michigan State University, East Lansing, MI, United States

**Keywords:** exercise, eHealth, mHealth, internet, adherence, compliance

## Abstract

**Background:**

The purported benefits of online physical activity interventions, in terms of reduced costs, high reach, and easy access, may not be fully realized if participants do not engage with the programs. However, there is a lack of research on modifiable predictors (e.g., beliefs) of engagement with online physical activity interventions. The objective of this brief report was to investigate if self-efficacy to engage at baseline predicted subsequent engagement behavior in an online physical activity intervention at post-baseline.

**Methods:**

Data (*N* = 331) from the 2018 Fun For Wellness effectiveness trial (ClinicalTrials.gov, identifier: NCT03194854) were analyzed in this brief report. Multiple logistic regression was fit in M*plus* 8 using maximum-likelihood estimation.

**Results:**

There was evidence that self-efficacy to engage beliefs at baseline positively predicted subsequent engagement behavior in the Fun For Wellness intervention at 30 days post-baseline.

**Conclusions:**

Some recommendations to increase self-efficacy to engage in future online physical activity intervention studies were provided consistent with self-efficacy theory.

## Introduction

Online physical activity interventions have both strengths and weaknesses. A clear strength of online interventions is that they can reach large numbers of targeted individuals at a low cost (1). However, providing access to an online intervention does not guarantee that those with access will engage with the intervention. Engagement with online interventions refers to the extent (e.g., amount, frequency, duration, depth) of usage and a subjective experience characterized by attention, interest, and affect (2, 3). Lack of engagement is a fundamental problem in online behavioral change intervention studies because it can lead to the ineffectiveness of the intervention, dropout from the study, and loss of follow-up (3, 4). Engagement can be relatively more problematic in online interventions which are often self-guided, compared to in-person interventions where participants are more closely supervised (5). Engagement may be an important factor in the effectiveness of online physical activity interventions (2–4). We note that some scholars use different terminology (e.g., compliance) to describe engagement.

In the online physical activity intervention setting, there is recent evidence that engagement may positively influence physical activity changes (6). Because of the importance, theoretically modifiable determinants of engagement with online interventions need to be highlighted, measured, analyzed, and discussed. However, although some studies examined predictors of engagement (e.g., demographics) in online physical activity interventions (7), there is a lack of research on modifiable predictors of engagement with these interventions. We believe that it is important to understand why some individuals engage with the online interventions while others do not (e.g., what predicts engagement). Specifically, investigating theoretically modifiable predictors of engagement may maximize the strengths of online physical activity interventions, contributing to the design and delivery of the online interventions (8).

According to self-efficacy theory (9), an individual's self-efficacy to engage with an online intervention at baseline may predict an individual's engagement with the online intervention at post-baseline. Self-efficacy refers to domain specific beliefs held by individuals about their ability to successfully execute differing levels of performance given certain situational demands (9). Online interventions may require different abilities to engage (e.g., navigating website pages, finding a time and place with little interruption), compared to in-person interventions (e.g., transportation to the place). Given the unique situational demands, the level of beliefs held by individuals about their ability to successfully engage can influence their engagement with the online format. Although there have been some indications that self-efficacy to engage may be associated with engagement with online interventions (3, 10), there is little longitudinal research that tested the predictiveness of self-efficacy to engage at baseline on subsequent engagement behavior in online physical activity interventions.

Fun For Wellness (FFW) is a self-efficacy theory-based online intervention aimed to promote well-being and physical activity by providing capability-enhancing learning opportunities to participants (11). The capability-enhancing learning opportunities in the intervention comprise 152 interactive and scenario-based challenges organized in the online environment by the acronym BET I CAN learning opportunities: *B*ehavior (e.g., developing goals), *E*motion (e.g., coping with negative emotion), *T*hought (e.g., creating a new story), *I*nteraction (e.g., interacting with other people), *C*ontext (e.g., identifying cues in the environment), *A*wareness (e.g., understanding themselves), and *N*ext steps (e.g., having a plan). For example, the behaviors learning opportunity guides how to develop practical goals and habits for regular physical activity and diet. Adults with obesity were targeted in the 2018 FFW effectiveness trial because there is consistent evidence that a majority of adults with obesity do not meet public health guidelines for physical activity, for example, 150 min per week of moderate physical activity (12). Broadly focused interventions for populations at-risk for a narrower health problem is an established practice in prevention science (13).

The objective of this brief report was to investigate if self-efficacy to engage at baseline predicted subsequent engagement behavior in an online physical activity intervention at post-baseline. The hypothesis was that self-efficacy to engage at baseline would positively predict engagement behavior in the FFW intervention at 30 days post-baseline—consistent with self-efficacy theory.

## Method

Data used in this brief report were collected in the 2018 FFW effectiveness trial (ClinicalTrials.gov, identifier: NCT03194854). The self-efficacy to engage data have not been reported in any previous publications. The engagement data were reported in previous publications to describe engagement with the FFW intervention (14–17). For example, objective data (i.e., task completion) and subjective data (i.e., user experience) indicated that many (but not all) participants moderately engaged with and enjoyed the FFW intervention (17). The data were analyzed with inferential statistics to investigate the new hypothesis in the present study. A summary of the 2018 FFW effectiveness trial is provided in this brief report so that readers do not need to consult the previously published papers. Then, key information about the present study is provided.

### The 2018 FFW effectiveness trial

The randomized controlled trial was a large-scale, prospective, double-blind, parallel group randomized controlled trial (11). Participants were remotely recruited through a health care panel recruitment company. There were five eligibility criteria: (a) ability to access the online intervention, (b) residency in the United States, (c) age between 18 and 64 years, (d) a body mass index (BMI) of ≥25.00 kg/m^2^, and (e) not concurrently enrolled in any intervention promoting well-being or physical activity. The age criterion encompasses adults aged 18–64, based on the typical retirement age in the United States (18). The BMI criterion covers both the overweight category (25.00–29.99 kg/m^2^) and the obese category (≥30.00 kg/m^2^), in line with many interventions targeting physical activity in populations with obesity (1, 19). Parallel randomization of eligible participants to either the FFW group (*n*_FFW _= 331) or the wait-list control group (*n*_wait−list _= 336) was performed via software code that was written to accomplish equal (i.e., balanced) allocations to the FFW and control groups (11). Data were collected through the intervention website at three time points: baseline (T1), 30 days post-baseline (T2), and 60 days post-baseline (T3).

Participants assigned to the FFW group were provided 30 days of 24 h access to the intervention from T1 to T2. There were four introductory challenges that focused on orienting participants to the intervention. Participants assigned to the FFW group had to complete the four introductory challenges to be able to have access to the subsequent 148 non-introductory challenges. Participants were not given any specific instructions regarding the non-introductory challenges to complete. The challenges completed by participants assigned to the FFW group were tracked by computer software. Participants assigned to the wait-list control group were given the access to the intervention after the data collection was closed. Readers are referred to main outcome results from the 2018 FFW effectiveness trial (14–16).

### The present study

Data only from participants (*M*_age_ = 44.02, *SD*_age_ = 11.06) assigned to the FFW group (*n*_FFW _= 331) were used in the present study because the focal predictor, self-efficacy to engage, and the outcome variable, engagement behavior, were not measured from the control group.

#### Predictors

After completion of the four introductory challenges, participants assigned to the FFW group were asked to respond to the following item: *How confident are you in your current ability to get yourself to complete at least 15 FFW post-introductory challenges within the next 30 days?* Completing at least 15 FFW post-introductory challenges was used to measure self-efficacy to engage in the 2018 FFW effectiveness trial, consistent with both substantive and methodological considerations from the 2015 FFW efficacy trial (20). The criterion included at least 15 FFW post-introductory challenges, in addition to the four introductory challenges. Briefly, this criterion was based on: (a) expert consensus (e.g., requiring more than 2 hours of interacting with FFW), (b) practical considerations (e.g., achievable yet demanding a meaningful level of engagement), and (c) methodological aspects (e.g., ensuring the presence of some compliers). A five-category rating scale structure was used for this item, ranging from *0 = no confidence* to *4 = complete confidence*, based on effective self-efficacy rating scale structures (21). Asking a participant at the onset of an intervention to report their self-efficacy to engage is in line with previous methodological research on predicting engagement (22).

Along with self-efficacy to engage as the focal predictor, previously proposed covariates from the FFW protocol were utilized in the analysis (11). This set of covariates resulted in 14 variables: female (0 = no, 1 = yes), Black (0 = no, 1 = yes), Hispanic (0 = no, 1 = yes), vocational or technical school (0 = no, 1 = yes), some college (0 = no, 1 = yes), undergraduate degree (0 = no, 1 = yes), graduate degree (0 = no, 1 = yes), married (0 = no, 1 = yes), part-time employment (0 = no, 1 = yes), full-time employment (0 = no, 1 = yes), retired (0 = no, 1 = yes), age, BMI, and income, consistent with relevant literature (23–26). The covariates were collected through self-reports at the eligibility screening or T1.

#### Engagement outcomes

In this brief report, engagement behavior refers to the extent of usage given that online interventions provide a unique opportunity to objectively measure users’ engagement such as the number of challenges completed during the intervention period. Measure of engagement behavior was concordant with measurement of self-efficacy to engage based on self-efficacy theory (9). Specifically, engagement behavior in the FFW intervention was measured by a dichotomous variable (0 = not completing at least 15 FFW post-introductory challenges by T2, 1 = completing at least 15 FFW post-introductory challenges by T2). This operational definition for engagement behavior was guided by the measurement of self-efficacy to engage: a participant's confidence in their current ability to complete at least 15 FFW post-introductory challenges within the next 30 days. A key tenet of self-efficacy theory is that self-efficacy is most predictive of behavior when concordance is high. In conjunction with the dichotomous engagement outcome, we incorporated the raw number of post-introductory challenges completed (i.e., continuous engagement outcome) into a supplementary analysis.

#### Data analytic approach

Multiple logistic regression was fit in M*plus* 8 using maximum-likelihood estimation (27). Type I error rate was set equal to.05. Two-tailed hypothesis tests were used in the analysis of the parameters (i.e., more conservative than one-tailed). Missing data were modeled under the assumption of missing at random (28).

## Results

Missing data ranged from 0.00% in age to 12.39% in self-efficacy to engage, with a total of 95.05% of the data observed (i.e., not missing). Descriptive statistics by engagement groups are provided in Table 1. A majority of participants (80.69%) engaged with the FFW intervention. In self-efficacy to engage, the average from the engaged group was higher than the average from the not engaged group: 3.39 vs. 2.89. In age, the average from the engaged group was lower than the average from the not engaged group: 42.39 years vs. 49.61 years. For gender, females were slightly more engaged than males: 70.94% vs. 29.06%. There was little difference by engagement groups in terms of other covariates (e.g., BMI). Post-hoc tests showed statistically significant differences in the mean of the focal predictor (i.e., self-efficacy to engage, *p *< .001) and in the proportions of occupational status (e.g., full-time employment, *p* = .002; retired, *p *= .016; income, *p* = .004). There were no statistically significant differences in the proportions of any demographic characteristics or education.

**Table 1:** Descriptive statistics by engagement groups.

	Not engaged (*N* = 56)	Engaged (*N* = 234)
Focal Predictor		
Self-efficacy to engage (*M, SD*)*	2.89 (1.07)	3.39 (0.77)
Demographic characteristics		
Age (*M, SD*)	49.61 (10.51)	42.39 (10.81)
Body mass index (*M, SD*)	31.67 (5.90)	30.57 (6.11)
Female (*n*, %)	35 (62.50)	166 (70.94)
Black (*n*, %)	7 (12.50)	33 (14.10)
Hispanic (*n*, %)	5 (8.93)	14 (5.98)
Married (*n*, %)	31 (55.36)	159 (67.95)
Education		
Vocational or technical school (*n*, %)	5 (8.93)	19 (8.12)
Some college (*n*, %)	13 (23.21)	37 (15.81)
Undergraduate degree (*n*, %)	18 (32.14)	91 (38.89)
Graduate degree (*n*, %)	7 (12.50)	54 (23.08)
Occupational status		
Part-time employment (*n*, %)	8 (14.29)	22 (9.40)
Full-time employment (*n*, %)*	27 (48.21)	162 (69.23)
Retired (*n*, %)*	10 (17.86)	17 (7.26)
Income (*M, SD*)*	88.27(198.62)	76.06(45.84)

Age was measured in years, and body mass index was measured in kg/m^2^. The asterisk indicates that there was a statistically significant difference between engagement groups when the type I error rate was set to.05.

Statistical tests of predictors in the multiple logistic regression model are provided in Table 2. The focal predictor was statistically significant: self-efficacy to engage, *β *= 0.58, *SE* = 0.18, *p* = .002. For each one-unit increase in self-efficacy to engage, the odds of being engaged increased from 1.00 to 1.79. Two covariates were statistically significant: age, *β *= −0.05, *SE* = 0.02, *p* = .01; female, *β *= 0.79, *SE* = 0.38, *p* = .04. For each one-unit increase in age, the odds of being engaged decreased from 1.00 to 0.95. For being female, the odds of being engaged increased from 1.00 to 2.21. No other covariates, such as BMI, statistically predicted the dichotomous engagement outcome. Additionally, a supplementary analysis revealed that self-efficacy to engage significantly predicted both the dichotomous engagement outcome (*β *= 0.59, *SE* = 0.18, *p* = .001) and the continuous engagement outcome (*β *= 11.14, *SE* = 3.55, *p* = .002). In summary, there was evidence that self-efficacy to engage at baseline (T1) positively predicted subsequent engagement behavior in the FFW intervention at 30 days post-baseline (T2).

**Table 2:** Results of the multiple logistic regression model predicting dichotomous engagement behavior in the FFW intervention by self-efficacy to engage and fourteen covariates.

Predictor	β	*SE*	*p*	Odd ratio	95% CI for odd ratio
Self-efficacy to engage	0.58	0.18	.002	1.79	[1.25, 2.57]
Age	−0.05	0.02	.01	0.95	[0.92, 0.99]
Body mass index	−0.01	0.03	.65	0.99	[0.93, 1.04]
Female	0.79	0.38	.04	2.21	[1.05, 4.63]
Black	−0.03	0.50	.96	0.98	[0.37, 2.59]
Hispanic	−1.02	0.61	.10	0.36	[0.11, 1.20]
Married	0.29	0.35	.41	1.34	[0.67, 2.65]
Vocational or technical school	0.25	0.64	.70	1.28	[0.37, 4.50]
Some college	−0.26	0.51	.60	0.77	[0.29, 2.07]
Undergraduate degree	0.09	0.52	.86	1.09	[0.40, 3.02]
Graduate degree	0.23	0.64	.72	1.26	[0.36, 4.39]
Part-time employment	−0.27	0.59	.64	0.76	[0.24, 2.41]
Full-time employment	0.42	0.51	.41	1.52	[0.56, 4.16]
Retired	−0.24	0.59	.69	0.79	[0.25, 2.51]
Income	−0.004	0.004	.24	1.00	[0.99, 1.00]

The dichotomous outcome variable in the model is engagement behavior in the FFW intervention (0 = not completing at least 15 FFW post-introductory challenges by Time 2, 1 = completing at least 15 FFW post-introductory challenges by Time 2). The predictors were measured at baseline and are adjusted for each other in the model. The continuous predictors were: self-efficacy to engage, age, body mass index, and income.

## Discussion

The purported benefits of online physical activity interventions, in terms of reduced costs, high reach, and easy access, may not be fully realized if participants do not engage with the programs. The objective of this brief report was to investigate if self-efficacy to engage at baseline predicted subsequent engagement behavior in an online physical activity intervention at post-baseline. As hypothesized, the results showed that self-efficacy to engage at baseline positively predicted engagement behavior in the FFW intervention at 30 days post-baseline. The theoretical mechanism for the focal predictor of engagement is discussed based on self-efficacy theory. Some recommendations to increase self-efficacy to engage in future online physical activity intervention studies are provided.

The findings of the focal predictor in the present study are consistent with self-efficacy theory, which may contribute to the development of future online physical activity intervention studies. The prediction of engagement can be explained by the mechanism where high judgments of self-efficacy may lead to increases in behavior both directly and indirectly through other behavioral predictors. To be specific, judgments of self-efficacy to engage could influence subsequent engagement behavior in interventions both directly and indirectly through outcome expectancies, goals, perceived barriers, and facilitators for the behavior. According to self-efficacy theory (9), individuals’ beliefs in their efficacy can be active producers that influence the courses of actions they choose to pursue. The role of self-efficacy could also be important in online physical activity intervention settings to improve users’ engagement. For many online physical activity intervention studies in which users are self-guided with the use of the Internet, relatively low engagement may be a natural and typical feature. This limitation can be addressed by modifying self-efficacy to engage because the engagement can be a function of self-efficacy to engage, as noted in the present study and self-efficacy theory.

In a future online physical activity intervention study, it would be worth exploring whether exposing participants to introductory tasks designed to increase self-efficacy to engage prior to exposure to the remaining tasks improves engagement behavior. In the 2018 FFW effectiveness trial, the introductory challenges were not specifically designed to increase self-efficacy to engage. The introductory tasks aimed to increase self-efficacy to engage can be based on the cognitive processing of diverse sources of efficacy information: mastery experience, vicarious experience, verbal persuasion, and physiological and affective states (9). For example, an introductory task can be guided by mastery experience such as practicing how to use an online intervention. Another introductory task can be guided by vicarious experience such as watching a vignette that shows a targeted individual (e.g., an adult with obesity) successfully engages with an online physical activity intervention. Researchers in online interventions have recommended using introductory tasks during which users become familiar with what can be asked of them during the interventions and demonstrate their commitment by completing the tasks (29). Introductory tasks in online physical activity interventions can be designed to increase participants’ self-efficacy to engage for the purpose of improving subsequent engagement with online physical activity interventions.

Regarding the covariates, the results showed that age statistically predicted engagement behavior in the FFW intervention at 30 days post-baseline. The age findings are contradictory to previous empirical or review research that focused on in-person interventions for adults with obesity (23–25). In-person interventions may require different efforts and commitments (e.g., ability for in-person communications), compared to online interventions (e.g., navigating website pages, self-guided use). In the context of online physical activity interventions, it may be suspected that younger users exhibit more engagement behavior compared to older users. However, the beta value for age was small (i.e., −0.05), indicating that the findings regarding age and engagement behavior in this study warrant further investigation in subsequent online physical activity intervention studies. Also, the results showed that being female increased the odds of being engaged. This result could be found by chance—or something specific to the FFW intervention—because findings of engagement difference by gender tend to be inconsistent (25).

Overall, the present study may provide a starting point to suspect that users could engage with online physical activity interventions if they believe that they can successfully engage with the interventions. It is recommended to increase self-efficacy to engage by using introductory tasks in online physical activity interventions, based on the cognitive processing of diverse sources of efficacy information. We are aware of at least one noteworthy limitation in the present study. The self-efficacy to engage was measured by one item because this was a pilot measure in the 2018 FFW effectiveness trial. Given the initial positive results reported in this manuscript, there may now be a need to develop a new scale with multiple items to more rigorously measure multi-dimensional self-efficacy to engage (e.g., amount, frequency, duration, depth), consistent with a multi-dimensional conceptualization of engagement (3). We believe that future studies should increase efforts to investigate a theoretical understanding of engagement and to incorporate the findings into the design and delivery of online physical activity interventions.

## Data Availability

The data analyzed in this study is subject to the following licenses/restrictions: the corresponding author may answer questions and/or requests regarding the dataset. Requests to access these datasets should be directed to seunglee@iastate.edu.
